# Bridging the Digital Divide for Rural Older Adults by Family Intergenerational Learning: A Classroom Case in a Rural Primary School in China

**DOI:** 10.3390/ijerph19010371

**Published:** 2021-12-30

**Authors:** Hao Cheng, Keyi Lyu, Jiacheng Li, Hoiyan Shiu

**Affiliations:** 1 Department of Education, East China Normal University, Shanghai 200062, China; chenghaophd@163.com (H.C.); 51204101061@stu.ecnu.edu.cn (H.S.); 2 Shanghai Municipal Institute for Lifelong Education, East China Normal University, Shanghai 200062, China; jcli@dem.ecnu.edu.cn

**Keywords:** digital society, digital divide, family intergenerational learning, home-school cooperation, rural older adult, China

## Abstract

Rural older adults often feel disconnected from the ever-expanding digital world. To bridge the digital divide, researchers have investigated the effectiveness of formal education and training offered by various social institutions. However, existing research highlights a critical shortcoming in these approaches: a lack of attention paid to rural older adults’ individual needs and interests. Based on the theories of post-metaphorical culture, endogenous development, home-school cooperation, and technology adoption and acceptance, this study implements a family intergenerational learning (FIL) project. FIL characterizes learning between grandparents and grandchildren within the household, suggesting a more practical and individualized strategy to help rural older adults gain digital literacy. By conducting a three-month FIL Project in a rural primary school class in China, the study employs a qualitative method to analyze learning records and interviews from 10 sets of participating grandparents and grandchildren. The analysis renders two critical findings on the effectiveness of the FIL Project for rural older adults. First, FIL can help rural older adults adapt into the digital world by (1) gaining knowledge about digital society, (2) improving their digital skills, (3) changing their lifestyles, and (4) understanding the integration between technology and society. Second, among grandchildren, FIL can cultivate an awareness of lifelong learning and their moral obligations to their grandparents. By illustrating this specific case, this study puts forward a new approach to help the older adults overcome the digital divide in rural areas.

## 1. Introduction

Worldwide, the proportion of older adults aged 60 or above is growing rapidly [1]. Understanding how they experience an increasingly digital society is pertinent to the quality of life for this population. The older people, who were used to a slow-paced and closely connected world [2,3,4], are dealing with a digital divide when engaging in interpersonal communication, accessing medical services, and acquiring essential information [5,6]. Existing research shows that adults over the age of 65 are particularly notable in their cautious approach to the online world [7,8,9]. These older adults users have less trust in the Internet and report higher levels of confusion by the amount of information online [8].

According to several studies, the digital divide reduces older people’s interests in and ability to absorb new knowledge, spurring feelings of anxiety, fear, and insecurity [10,11]. This trend has become more alarming as the influence has broadened and deepened [12,13]. By contrast, if older adults had a mastery of digital technology, their mental health state would be improved through a reduction of loneliness [14], depression rates [15], and cognitive decline [16].

The question on how to motivate older adults to use technology and help them overcome the fear it creates has attracted much attention since the 1980s. For example, in 1982, the United Nations organized the First World Assembly on Ageing and formulated the Vienna International Plan of Action on Ageing [17]. It recommended countries to actively help older adults to solve technical and digital issues. In 1999, the European Union further discussed strategies to assist older adults with adapting to and engaging with the Internet age. In 2002, Active Aging: From Evidence to Action [18], published by the World Health Organization, conceptualized active ageing from dimensions of health, participation, and security and proposed pragmatic approaches. It is evident that nations and international organizations believe in assisting older adults in bridging the digital divide as a critical component of future social progress.

China, the largest developing country, with 264.02 million individuals aged 60 and above, is a critical part of the solution to the issue of digital divide. In 2020, the State Council of China issued the Implementation Plan for Practically Solving the Difficulties of Older Adults in Using Intelligent Technology, intending to improve older citizens’ sense of acquisition, happiness, and security in the information society [19]. Compared with urban older adults, however, those living in rural areas are facing more serious challenges from the digital divide [20]. There is less use and promotion of intelligent elderly care or information technology services, resulting in a more systematic digital gap between older adults in urban communities and those in rural areas [21,22,23].

Thus, helping older people gain digital literacy skills is beneficial not only for their individual well-being but also for the harmony and equity of society [9,24]. One research work investigated the role of the library in providing information literacy training for rural older adults from the perspective of rural revitalization [25]. However, the results shown were not satisfactory in meeting the needs of older adults for digital learning. Most other existing studies explore how to improve the digital literacy of urban older adults; therefore, the understanding of and approaches to the issue among rural older adults are lacking in China [26,27,28].

In rural areas of China, 35.98% of adults aged 60 and above live in a multi-generational household with their children and grandchildren [29]. Another report shows that 96% of left-behind children are taken care of by their grandparents [30]. This phenomenon is not only a result of cultural tradition but also state policies that created barriers for internal migration [31]. Some studies suggest that family, peers [32], and intergenerational relationships [33] may play a more prominent role in bridging the digital divide for older adults. Moreover, schools can help motivate students/families to interact with their grandparents [34,35]. In designing an effective approach to intervene with the digital divide for rural older adults, we ask the following: how can the involvement of rural schools contribute to reducing the digital divide for rural older adults?

The research team selected a rural primary school in East China as the field site to implement a family intergenerational learning (FIL) project (hereafter referred to as the FIL Project). The duration of the FIL Project was three months, and student participants were encouraged to teach their grandparents digital knowledge and practices using digital devices. The paper explores the implementation of the FIL Project by the rural primary school and evaluates its outcomes.

## 2. Literature Review

Researchers have proposed different solutions to solve the digital problem for older adults. Different subjects, actors, and sites have been studied in understanding the effectiveness of these solutions.

### 2.1. Top-Down Service from the National Government

Governmental support plays a dominant role in bridging the divide. For example, to meet the rising learning demands for information literacy and encourage social integration, the Korean government has developed free online education systems or training platforms, such as Learning Country, which is freely accessible to its citizens [36]. The government of Spain executed media literacy and e-learning projects to help older citizens integrate into the digital era. However, due to unequal economic growth across regions, many older adults lacked the essential access to the Internet [37]. This digital divide as a result of economic inequality is also present in Hong Kong. Although large-scale training can meet the needs of older adults, these particularly vulnerable groups continue to struggle with the issue of access [38]. In the United Kingdom, the government provides Internet connection to older adults, yet the resource is not useful sometimes because of insufficient skills and a mistrust of the Internet [39]. The Australian federal government has been planning to construct the National Broadband Network in rural regions since 2009. However, the new administration postponed the project in 2013 due to financial constraints [40]. Government-level participation in bridging the digital divide is usually inadequate to address the issue holistically, continuing to marginalize older adults in a digital world.

### 2.2. Research-Based Intervention Programs from Researchers/Scholars

Universities frequently conduct “vacuum” experiments (in contrast to social experiments) to investigate ways to assist older individuals in bridging the digital divide. However, there are numerous issues with this approach. In Computing Senior Workshops, for example, university researchers recruited elderly participants with an interest in studying IT. However, due to challenges in adapting to computer equipment in the laboratory, older adults are under-trained [41]. Students at the University of Valladolid taught standardized rather than individualized IT-related content, but a lack of follow-ups failed to provide comprehensive evaluations of the programme [42]. The aforementioned research illustrates the challenges of executing university-proposed solutions in bridging the digital divide among older adults.

### 2.3. Training Projects in Community Schools and Geriatric Universities

Older adults frequently benefit from educational activities offered by community schools, geriatric universities, and other activity centers [43]. In 2014, at the Same Senior Living Center in the United States, 72 older adults were digitally trained [44]. However, as enrollment requirement specified access to a personal tablet computer, many older people without an electronic gadget were excluded [45]. Other research studying community centers found that seniors often benefit from such training with access to computers, opportunities for social networking, and the navigation of recreational websites [46]. Scholars often argue that standardized training often fails to be helpful because of the mismatch between training content and real-life encounters in the digital world [47]. However, an entry requirement is often suggested by community-based training. For the effectiveness of such programs, instructors often admit older adults who can better engage based on their socioeconomic background, thereby excluding older adults for whom the training may be more necessary.

### 2.4. Support Programs from Non-Governmental Organizations (NGOs) or Private Institutions

Social institutions and the commercial sector can also contribute to the supply of digital learning resources for older adults. There have been a few studies suggesting that computer training at the public library can help improve digital literacy for older adults [48]. However, due to the limited number of training seats and the uniformity of training material, the training content is incompatible with the needs of older adults, making it impossible to meet the individualized digital training needs of all older adults [49,50]. Some researchers suggest using interactive TV shows [51] or programs such as Research Institute and After-School Programs to facilitate closer connections with the digital world for older adults [36]. Overall, these services are essential for older adults to maneuver in the increasingly digital society, but the same issue associated with standardization in publicly accessible materials and the high cost of private trainings hinder marginalized elderly communities from gaining digital literacy.

### 2.5. Intergenerational Learning

As shortcomings of these initiatives become more evident, the academic community critiques the excessive reliance on formal education and training. Obviously, macro-level interventions increases the financial burden on the state and society, failing to meet standards for sustainable development. To promote social inclusion, some academics have created a method of intergenerational learning, a method of informational learning that has been viewed as a critical improvement to bridging the digital divide for older adults.

Currently, there are two types of intergenerational learning. The first is in the form of peer support and learning among older adults of the same age, known as ‘peer tutors’. The process involves pairing up older adults with more IT experience with those with less [52]. For example, OldKids, a training institution based in Shanghai, China, offered computer courses that were taught by older adults with relevant skills [53]. The second kind of intergenerational learning engages young people to serve as ‘Intergenerational Mentors-Up’ to educate older generations about IT [54]. For instance, at a small town in the south of The Netherlands, university students educated senior citizens on how to use the Internet via social events and volunteering, which is critical for their social integration [55]. Additionally, in Italy, grandparents and grandchildren engaged in short-term digital contacts to promote cultural transmission [56]. However, older adults have a harder time achieving sustainable development because intergenerational learning is generally short-term and discontinuous.

While highlighting the benefits of these methods, scholars have two major criticisms: first, that the intergenerational learning programmes described above are one-time training sessions, activities, or courses, making it difficult to develop into a long-term model with sustainable qualities [57]; second, that there is a lack of longitudinal evaluation of intergenerational learning programmes and older adults’ digital literacy [54].

Although the cost of implementing intergenerational learning on a large scale is lower than state-sponsored programmes, universities, community colleges, and education centers, this research finds that there are at least two drawbacks. First, it tends to see older adults as the sole subject, while the fact that grandchildren are seen as tools to impact the subject disregards their involvement in projects. Second, most field sites are based in metropolitan areas, suggesting that the formal features of these learning programs fail to address the informal learning process of technological knowledge. Therefore, in order to bring IT to the disadvantaged groups of older adults, this study seeks to explore how schools and families can encourage rural older adults to gain digital literacy.

## 3. Theoretical Basis

### 3.1. Endogenous Development

In 1975, Dag Hammarskjöld proposed the theory of Endogenous Development in the United Nations Report “The Future of the World”. In Fernando’s view, if development is understood as the emancipation and comprehensive development of the human being, then this so-called development can only be achieved by the internal forces of society [58]. As illustrated in the literature review above, current initiatives to assist older adults in bridging the digital divide have a number of flaws: (1) a focus on external and generalized education has led to a separation between training content and real-world applications; (2) not all older adults have the opportunity and ability to participate in training; (3) the high cost of education has increased the financial burden on older adults and states. Based on the insights of Endogenous Development, this paper argues that addressing the internal demands of older adults can remedy many of the disadvantages of external training.

### 3.2. Prefigurative Cultures

In Culture and Commitment: A Study on the Generation Gap, Margaret Mead, an American sociologist in cultural change and human interactions, distinguishes postfigurative, configurative, and prefigurative cultures. Postfigurative culture refers to the process by which young children learn from their parents, grandparents, and other adults. Cofigurative cultures describes the peer learning process for children and adults. Prefigurative culture is one in which adults learn from their children as well as from their ancestors and peers [59]. In the digital age, younger people have more information knowledge and skills than older adults. Thus, the prefigurative culture provides the basis for the hypothesis of this study that learning from younger generations may be an efficient way for older adults to facilitate the digital divide.

### 3.3. Home–School Cooperation

The Overlapping Spheres of Influence model recognizes that there are some practices that family, school, and community conduct separately and that there are others that they conduct jointly in order to influence the growth and learning of the child [60,61]. The model has become the theoretical and practical basis for many countries to promote a home–school cooperative education process since the end of the 20th century.

Despite the prevalence of multi-generational co-habitation in China today, family members have paid little attention to intergenerational learning. This study reveals that collaboration between home and school is a critical external force supporting the successful implementation of intergenerational learning in families. By repeatedly performing intergenerational learning at home, school teachers can contribute to the improvement of family dynamics between grandparents and grandchildren, facilitating grandparents to acquire a more proficient understanding of the digital world.

### 3.4. Theories about Technology Adoption and Acceptance

Some theories or models related to technology acceptance—including the theory of reasoned action (TRA) [62], the technology acceptance model (TAM) [63], and the unified theory of acceptance and use of technology (UTAUT) [64]—reveal additional layers of older adults’ motivations to use technology. Of all the theories, the Technology Acceptance Model (TAM) [63] is considered the most influential theory for describing an individual’s acceptance of information systems [A]. Davis assumes that an individual’s information systems acceptance is determined by two major variables. One is perceived usefulness (PU), which reflects the value that a person may add to his work performance by means of external tools such as information technology. The other is perceived ease of use (PEOU), which reflects the degree to which a person finds it easy to use a specific system [63,65,66]. With an aging population and rapid technological innovation, Ke Chen and Alan Hoi Shou Chan put forward a new senior technology acceptance model (STAM) that takes into consideration the unique capabilities of older adults [67]. The model argues that personal characteristics (age, education, gerontechnology self-efficacy and anxiety, and health deficiencies) and environmental facilitating supports (accessibility, assistance, and guidance) has more predictive value than attitudinal factors (usefulness and ease of use) for predicting gerontechnology usage behavior. Esther Hargittai and other scholars also find that people from disadvantaged backgrounds have lower internet skills than those from more privileged backgrounds [9,32,68,69]. Thus, the needs and abilities of older adults in digital system design must be taken into account to better promote their engagement with the digital world [70,71,72,73]. Based on these theories and research, a more in-depth and experiment-based exploration on a strategy that promotes rural older adults in China is necessary to better customize education programs for older adults to gain equal share in this digital world.

## 4. Methodology

Our study aims to explore the effectiveness of the FIL Project based on a classroom case in a rural primary school in China. It was an interpretive inquiry, which follows an exploratory and emergent qualitative research design where researchers select a data analysis strategy that best fits the information stemming from participants’ disclosures [74]. Qualitative studies typically use purposively selected samples (as opposed to probability-driven samples), which seek a diverse range of “information-rich” sources [75] and focus more on the quality and richness of data rather than the number of participants. Saturation (i.e., obtaining a comprehensive understanding by continuing to sample until no new substantive information is acquired) is a core guiding principle to determine sample sizes in qualitative research [76,77]. Based on the nature of qualitative methodologies, 10 sets of grandchildren and grandparents who were deeply involved in the FIL Project and could provide appropriate, useful, and adequate data to effectively answer the research questions [78,79] were labeled as the research sample. This article will illustrate: (1) the implementation of the FIL Project; (2) the research process; and (3) data analysis.

### 4.1. The Classroom in a Rural Primary School

Based on our research question, we recruited 43 third-grade students from a rural primary school as the research group for two main reasons. First, all 33 students in the class lived with or close to their grandparents. Specifically, 6 students lived near their grandparents, 24 students lived with both their parents and grandparents, and 4 students were left-behind children (living only with their grandparents). Therefore, the FIL Project could cover most families in the class. Second, the head teacher Tu S.L. (anonym), who was the key person in charge of the class and responsible for communicating with families of the class, was motivated to participate in the FIL Project. Thus, it was ensured that the FIL Project would run smoothly.

### 4.2. Research Process

#### 4.2.1. Online Questionnaire Survey for Selecting Family Intergenerational Learning (FIL) Project Participants

In this study, there were three inclusion criteria. First, only those living with or nearby their grandchildren were included. Second, participants had to have basic reading and writing skills (not illiterate). Third, participants were personally motivated to participate. On 27 June 2021, Tu S.L. was in charge of recruiting participants, delivering the paper questionnaire to 33 students, and asking them to fill it out with their grandparents. If grandparents could not write, children wrote down what their grandparents said word for word. All questionnaires were collected in the next day.

According to the poll, 24 sets of grandparents and grandchildren were interested in the FIL Project. Based on the descriptive statistics on the 24 sets, the grandparents were interested in learning how to use digital devices (e.g., mobile phones, computers, smart watches, etc.) to search for health information, check health codes (“Health Code” is one of the most popular mobile applications that has been used during COVID-19 pandemic in China. It acts as an e-passport that records the real-time personal health condition of the user and is organized by the government. For older adults, learning to use Health Code and some other mobile apps is vital for them to adapt the new but normal life), pay bills, and use social platforms such as WeChat and QQ (two popular social platforms in China for messages, phone calls, group chats, and sharing updates with their social circles) for socialization and communication.

#### 4.2.2. Conducting the Three-Month FIL Project and Collecting Learning Records

After obtaining informed consent from all participants, the authors worked with Tu S.L. to execute the 3-month FIL Project from 14 July to 14 October. Specifically, the first author explained the research purpose to the students and their grandparents and obtained their oral consent of participating in this study. We also obtained written consent from Tu S.L. and the students’ parents.

**Figure 1:**
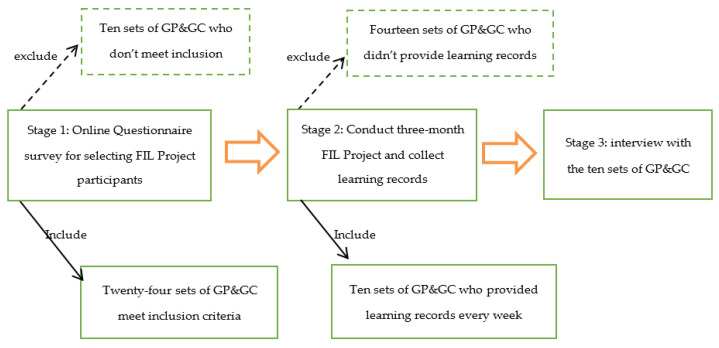
Research process and sampling stages.

The grandchildren were encouraged to teach their grandparents digital knowledge and skills based on their personal interests and needs. In order to track the intergenerational learning process, as well as participants’ feelings and experiences, the authors set up a WeChat group. Members in the group included Tu S.L., the first, second, and third authors of this study, and 24 sets of GP and GC. The teacher would communicate with participants daily to ensure that older adults’ learning needs were met. On a weekly basis, grandchildren and grandparents were asked to complete the Learning Record Form together and submitted the form to the first author. The form included three questions: (1) Where, when, and how often did you teach your grandparents? (2) What digital knowledge or digital skills did you teach your grandparents? (3) How did you and your grandparents feel during this process? At the conclusion of the study, 10 sets of GP and GG provided learning records to the first author accordingly. Those who failed to complete the form reported reasons of thinking that this research was meaningless or a lack of time. In order to understand how grandchildren helped their grandparents gain digital literacy and what the values of FIL Project are, these 10 pairs were invited for follow-up interviews (see Figure 1).

#### 4.2.3. Conducting Online Interviews with Grandchildren and Grandparents

From 18 October to 28 September, the authors carried out online interviews with 10 sets of GP and GC. Demographic information on these sets of GP and GC is presented in Table 1. The interview questions for grandparents included three open-ended questions: (1) “GPQ1-Have your learning needs in the digital society been met? Why?”; (2) “GPQ2-Have the digital knowledge or skills you gained during this project helped you adapt better to the digital society? Why?”; (3) “GPQ3-What experiences and feelings did you have while participating this project?”. The two main interview questions for grandchildren were “GCQ1-How did you teach your grandparents?” and “GCQ2-What experiences and feelings did you have in this project”. Sometimes, the researchers would offer appropriate guidance or propose follow-up questions to obtain an informed response. For example, when asking GPQ3, the grandparents would be encouraged to share stories to improve the researchers’ understanding.

### 4.3. Data Analysis

Qualitative data analysis comprises three stages: (1) a transcription, (2) an identification, examination, and interpretation of patterns and themes in textual data, and (3) a formulation of responses to research questions based on patterned themes [80,81,82]. In the first stage, the first author and second author transcribed all learning record forms and interview records and created a preliminary database [83,84]. To guarantee validity of the coding process, a code book was discussed and agreed upon by the first, second, and third authors. The first author and second author then coded the data for certain words or content and identified patterns relevant to the research questions [78,85]. The authors coded independently and then cross-checked their analysis. When conflicts arose in the coding results, the third author with more research experience resolved them. The purpose of the third stage was to generate research findings on the significance of the FIL Project for older adults and compare the findings with existing research [86,87].

## 5. Findings

Based on this specific case study, this paper presents two main findings: First, the FIL Project helped older adults to gain digital literacy, which was manifested in gaining knowledge about digital society, improving digital skills, changing lifestyles, and understanding the integrated relationship between technology and society. Second, the FIL Project also contributed to the grandchildren’s awareness of lifelong learning and of their moral obligations to their grandparents. The latter is important for implementing this project and creating better intergenerational relationships.

### 5.1. Helping Rural Older Adults: Bridging the Digital Divide in Four Ways

#### 5.1.1. Gaining Knowledge about Digital Society

As mentioned above, in a society where technology progresses at an exponential rate, rural older adults often lack knowledge about the digital society. When asked the questions GPQ1 and GPQ2, most of the rural older adults mentioned that participating in this project helped them gain more knowledge about digital society and maintain an active engagement in life and society.

As some older adults said, the FIL Project provided a deeper understanding of digital society.

“I think that the speed of social development is too fast. I do not understand many new things in this era. Under the guidance of Teacher Tu S.L., my grandson introduced me to what is digital and smart, which made me feel that this is a world of virtual networks, the Internet, and information are prominent features of this society.”

(Du H.X.’s grandfather).

Some older adults learned the significance of information technology from the FIL Project:

“Most of my life has been in a non-digital and non-intelligent era. I used to think that the digital network was just a means of entertainment for young people. However, FIL broke my understanding of the digital society, and I gradually learned that information technology plays an important role in daily life, and even cannot be replaced on some occasions.”

(Zhang F.X.’s grandfather).

Some older adults gained more knowledge about different kinds of information and communication technologies.

“It is a pity for me that I did not finish primary school so that I could not adapt to the huge changes in the digital society. My granddaughter, as a digital native, taught me about digital information and virtual networks, such as the difference between mobile payment and cash payment.”

(Zhu. H.X.’s grandfather).

#### 5.1.2. Improving Digital Skills

Compared with their grandchildren, rural older adults were exposed to these new technologies late in their lives and have had to learn new skills to use them. By analyzing the learning records and interview data, most of the grandparents mentioned that they improved various digital skills during the FIL Project in the following aspects.

First, some older adults learned how to make electronic payments on mobile phones, tablets, and computers. He J.F.’s grandmother reported that this was useful.

“In the past, my son helped me register for medical treatment and pay taxi fares on digital devices. To be honest, I want to learn information technology to do the above things by myself. With Tu S.L.’s encouragement, my grandson taught me how to do it. Now I am able to use Alipay, WeChat, and other apps to make payments. It is really useful for the older adults like me.”

(He J.F.’s grandmother).

Second, some older adults learned how to communicate with their friends and relatives via social platforms. It was particularly important for them to cope with anxiety during the COVID-19 period.

“Face-to-face interpersonal communication has become extremely difficult during the COVID-19 pandemic. To help me meet my social needs, my grandson taught me to use the video and voice functions of WeChat to keep in touch with relatives and friends. I have realized that information technology plays an important role in daily life.”

(Zhu Yule’s grandfather).

Third, some learned to use digital devices for entertainment purposes.

“I became a lonely old person because my grandchildren went to school and my son with his wife went to work. It is hard to maintain my attention on repetitive TV programs. Fortunately, my grandson patiently taught me how to use the phone to browse news and play games, which added much happiness to my boring and lonely life.”

(Zhang R.Y.’s grandmother).

#### 5.1.3. Changing Lifestyles

Related to the previous findings, some rural older adults formulated a new lifestyle and learned about the relationship between learning and lifestyle by participating in the FIL Project.

As some rural older adults said, the FIL Project provided them with a platform to learn about information technology and profoundly updated and changed their previous lifestyle.

“Encouraged by Teacher Tu S.L., my grandson taught me many new things in this era, such as playing chess electronically, learning via online courses, and using mobile phones to monitor the running time and speed while doing exercises. As a result, my life has become richer and more scientific. Moreover, I gradually adopted a more positive attitude towards life and adapted to a lifestyle befitting of a digital society.”

(He J.F.’s grandmother).

Some rural older adults claimed that they lived a more “modern” life than they did before.

“In the past, my lifestyle was dominated by traditional face-to-face communication, reading paper books, and going to the marketplace to buy groceries. By learning with my granddaughter, I can read newspapers on my notebook computer and buy clothes and other daily necessities on my tablet computer now.”

(Xu X.Y.’s grandfather).

In addition, some rural older adults gradually learned about the relationship between learning and life, and expect to live more actively through lifelong learning.

“I have never been to school and have no strong learning needs. However, FIL has changed my understanding of learning. Recently, I realized the value and importance of learning for creating a better life. Therefore, from now on, I will choose to live a meaningful lifelong learning life with the help of my granddaughter.”

(Wu M.C.’s grandmother).

#### 5.1.4. Understanding the Integration between Technology and Society

Based on the above changes in knowledge, skills, and lifestyle, some rural older adults further developed their views on the relationship between technology and society.

By participating in the FIL Project, some rural older adults became aware of the close ties between individuals, technology, and the digital society.

“I used to think that the relationship between information technology and our real life was completely separate and that they were two opposing worlds. Family intergenerational learning fills my daily life with numbers, information, and technology. Therefore, I think smart phones, tablet computers, and electronic watches are closely related to each of us.”

(Wang Y.X.’s grandmother).

With the help of their grandchildren, some older adults learned the diversified functions of information technology and discussed the symbiotic relationship between information and technology rather than separation.

“In the past, I thought that using information technology was a necessary tool for young people to work and study and that older adults need not pay much attention. However, since my granddaughter taught me about electronic payment and online communication, I have a new understanding of the relationship between technology and society, which is not only coexistent but also intertwined.”

(Zhu. H.X.’s grandfather).

Some older adults understand the survival and developmental crisis of the digital society in depth, a crisis in which those who will not continue to learn are eliminated by the information society.

“FIL makes me feel that, if a person does not learn information technology, then they will be abandoned in this digital age. With the rapid updating of information technology, learning to use mobile phones and operate computers has become necessary for everyone. FIL not only strengthened the relationship between my granddaughter and me but also prompted me to have a sense of integration between technology and life.”

(Lu Y.X.’s grandfather).

### 5.2. Helping Grandchildren: Promoting an Awareness of Lifelong Learning and Moral Obligations to Grandparents

When asked the questions GCQ1 and GCQ2, most grandchildren mentioned that they were aware of the importance of lifelong learning in well-being and health.

“Since I taught my grandmother how to use the mobile phone to pay and chat with friends with WeChat, I felt that everyone needs lifelong learning to adapt to the rapidly developing and changing digital society. From now on, I will never be too old to learn like my grandmother.”

(He J.F.).

“Teaching grandfather to check the health code and record the amount of exercise he performs by mobile phone has made me understand the value and power of lifelong learning, which helps us to face a complex and changeable society and life smoothly.”

(Zhu. H.X.).

In addition, some grandchildren mentioned that they not only learned moral knowledge in the classroom as usual, but more importantly, carried out moral practices in their daily lives by participating in the FIL Project. They felt this project contributed to their moral development in many respects.

For example, one student said it made him realize that, as a grandchild, he also had a responsibility to help older adults.

“My parents and teachers have always taught me to be a person who is honest, polite, and respectful to older adults. With the development of intergenerational learning, my teacher encourage me to teach my grandmother to use the mobile phone, which made me further understand that it is my duty as a grandchild to help grandparents bridge the digital divide.”

(Wu M.C.).

“Usually, in school, we often learn about how to respect older adults. But in daily life, it is grandparents who take care of me. I take a lot from them, but never ‘give’ them anything. Because of this project, I understand that I can actually do a lot of things for them and care more about them.”

(Du H.X.).

Some students mentioned that, in this FIL Project, they realized their grandparents were vulnerable groups in a digital society, so they understand more about their grandparents and have become more willing to care for and help them.

“I never thought digital skills were so important to my grandfather. I always thought older adults didn’t need them. In fact, in life, he needs to pay for things on his smart phone, check health codes, and make video calls. Those skills help him better adapt to the society. Yes, I realized that he was excluded from the digital society. I need to give him more assistance, just as he always cares about me.”

(Zhu H.X.).

## 6. Discussion and Limitations

We conclude with three special features of the FIL Project, educational training, and intergenerational learning outside the family, to explain why it can be used to help rural older adults overcome the digital divide in China.

First, the absence of exclusion criteria based on education level or SES in the FIL Project implies that more rural older adults can be reached. In terms of learning topics, training prescribed by community centers, universities, and learning centers for older adults suffers from limited educational space [48] and instructor shortages [49]. When evaluating training efficiency and learning quality, older adults with a higher academic level, younger seniors, and urban older adults are preferred [44,45]. The exclusion of underprivileged and undereducated older adults may lead to the reproduction of class inequality [38]. In contrast, the FIL Project expands the eligibility criteria, granting learning opportunities for any rural older adults living with or close to their grandchildren to participate. The improved feasibility and convenience is a crucial advantage for the FIL method.

Second, the FIL Project empowers rural older adults with greater self-autonomy in choosing which digital skills to acquire and the approaches taken for learning. At public institutions and schools, much of the teaching content is pre-designed [36,39]. Materials do not necessarily meet the individual learning needs for older adults [37,40,48]. In the FIL project, to some extent, the digital learning content and goals are co-created by the grandchildren and grandparents. As the family took charge, the content and delivery remained flexible and individualized.

Third, to some extent, the FIL Project does not require expansive learning spaces or complicated learning materials. The only essential learning equipment is an electronic device, such as smartphone or computer. The cost of promoting public formal education and training is often a barrier [36,37,40]. Private educational institutions have boosted consumer spending, putting an extra strain on the financial burden of older adults [53]. With an aim to reduce costs, the FIL Project taps into the potential of school-family education. In the long term, the potential for large-scale, government-sponsored implementation is not associated with a very burdensome cost.

While this qualitative study primarily seeks a deep understanding of a specific research question, its generalization still needs to be considered [88,89,90]. Our research team have tried to promote FIL Project in other rural areas in China. A related study carried out in seven primary schools (four of which were rural primary schools) showed the FIL Project had a positive impact on the development of both the children and the older adults [34]. In an upcoming handbook of family intergenerational learning written by the first author and the third author, further studies on four rural primary schools reveal how the FIL Project helps older people bridge the digital divide and promotes other aspects of their development in detail [91]. To some extent, FIL Project has been successfully promoted in some rural areas of China, and we believe that it may also be carried out in other areas of the world with similar cultures and conditions.

Within the scope of the research, some limitations need to be considered. First, although this study presents a new effective approach to help rural older people overcome the digital divide, the widespread application of the approach is bound by a specific context. Factors such as a high rate of multi-generational cohabitation in Chinese rural areas and cooperative relations between primary schools and universities are necessary to implement this approach. Second, research on technology interventions among rural older adults has shown that the environment (e.g., the social atmosphere of FIL) may be confounded with family intergenerational learning itself to produce significant changes from an intervention. In this regard, the responses given by grandparents may be biased due to their positive interactions with their grandchildren (e.g., the socialization with other family members). Third, to understand how training sessions and interactions between grandparents and grandchildren actually occur, field observations are necessary. Fourth, this study has focused more on positive effects of the implemented interventions rather than the potential negative effects; therefore, a future study may be necessary.

## 7. Conclusions

The digital divide between older adults and the rest of the population is a worldwide issue. Conducting the 3-month FIL Project, our study explored a feasible approach to increase the access to and the use of technology among rural older adults in China. After participation in the project, rural older adults (1) gained knowledge about the digital society, (2) improved digital skills, (3) changed their lifestyles, and (4) understood the integrated relationship between technology and society. Their grandchildren also cultivated an awareness of lifelong learning and their moral obligations to grandparents.

## Figures and Tables

**Table 1:** Basic information about grandchildren and grandparents.

NO.	Grandchildren (Gender, Age)	Grandparents (Age, Education)
1	Xu X.Y. (f, 10)	Xu X.Y.’s grandfather (65, primary)
2	Du H.X. (m, 10)	Du H.X.’s grandfather (73, semiliterate)
3	Wang Y.X. (m, 10)	Wang Y.X.’s grandmother (60, junior high school)
4	Zhang F.X. (m, 10)	Zhang F.X.’s grandfather (66, primary school)
5	Zhu Y.L. (m, 10)	Zhu Y.L.’s grandfather (68, primary school)
6	Zhang R.Y. (f, 10)	Zhang R.Y.’s grandmother (69, semiliterate)
7	Wu M.C. (f, 10)	Wu M.C.’s grandmother (60, semiliterate)
8	Lu Y.X. (f, 10)	Lu Y.X.’s grandfather (70, primary)
9	He J.F. (m, 10)	He J.F.’s grandmother (59, junior high school)
10	Zhu. H.X. (f, 10)	Zhu. H.X.’s grandfather (73, semiliterate)

f = female; m = male; all names that appear in this article are pseudonyms. In this study, “semiliterate” is used to describe older adults who did not attend school or complete primary school, but are able to read and write at an elementary level.

## Data Availability

The datasets generated during the current study are not publicly available but are available from the corresponding author on reasonable request.
